# Retrospective Comparison of the Effectiveness and Safety of Ceftriaxone 1 g Twice Daily versus 2 g Once Daily for Treatment of Aspiration Pneumonia

**DOI:** 10.3390/antibiotics11080983

**Published:** 2022-07-22

**Authors:** Hideo Kato, Mao Hagihara, Yoshihiko Morikawa, Nobuhiro Asai, Hiroshige Mikamo, Takuya Iwamoto

**Affiliations:** 1 Department of Pharmacy, Mie University Hospital, Tsu 514-8507, Japan; yoshihiko5225@clin.medic.mie-u.ac.jp (Y.M.); taku-iwa@med.mie-u.ac.jp (T.I.); 2 Department of Clinical Pharmaceutics, Division of Clinical Medical Science, Mie University Graduate School of Medicine, Tsu 514-8507, Japan; 3 Department of Clinical Infectious Diseases, Aichi Medical University, Nagakute 480-1195, Japan; hagimao@aichi-med-u.ac.jp (M.H.); nobuhiro0204@gmail.com (N.A.); mikamo@aichi-med-u.ac.jp (H.M.); 4 Department of Molecular Epidemiology and Biomedical Sciences, Aichi Medical University Hospital, Nagakute 480-1195, Japan

**Keywords:** aspiration pneumonia, ceftriaxone, *Streptococcus pneumoniae*

## Abstract

Although a 2 g once daily administration of ceftriaxone remains the standard dosing regimen for the treatment of aspiration pneumonia, there are no studies to investigate the optimal dosing method. Hence, we retrospectively evaluated the effectiveness and safety of 1 g twice daily versus 2 g once daily administration of ceftriaxone in adult patients with aspiration pneumonia. Patients who received ceftriaxone for the treatment of aspiration pneumonia between 2015 and 2021 were included in this study. Clinical responses, inflammatory markers, and incidence of adverse events after completion of ceftriaxone therapy were investigated. In total, 33 patients received 1 g twice daily (group 1) and 28 received 2 g once daily (group 2) ceftriaxone for the treatment of mild-to-moderate aspiration pneumonia. Compared with that of group 1, group 2 demonstrated significantly improved clinical responses (group 1 vs. group 2, 84.8% vs. 100%, *p* = 0.0316). Although the safety profile was not significantly different between the two groups, the incidence of choleliths during ceftriaxone therapy in group 1 was higher than that in group 2 (31.3% vs. 9.1%, *p* = 0.174). Therefore, a 2 g once daily administration of ceftriaxone appeared to be a simple regimen adequate for the treatment of inpatients with mild-to-moderate aspiration pneumonia, which might not be heavily involved by anaerobes.

## 1. Introduction

Aspiration pneumonia is a crucial health issue associated with high mortality and long hospitalization periods in an aging population [1]. Moreover, the mortality associated with aspiration pneumonia is gradually increasing worldwide and the mortality is reported to be 21% [2]. Therefore, the administration of appropriate antimicrobials with activity against the expected causative bacteria is important to obtain a better prognosis in patients with aspiration pneumonia.

Aspiration pneumonia is often caused by bacteria such as Streptococci and Haemophilus [3]. Therefore, ceftriaxone, which has great antimicrobial activity against these pathogens, is frequently prescribed for its treatment [4]. However, despite its widespread use in adult patients with aspiration pneumonia, an optimal dosing regimen of ceftriaxone has not been clarified for adult patients with aspiration pneumonia.

Ceftriaxone is a third-generation cephalosporin with a broad spectrum and high tissue penetration [5]. In addition, unlike other cephalosporins, ceftriaxone exhibits very high protein binding (85–95%) in a concentration-dependent manner and provides a long plasmatic half-life of 8 h [6]; therefore, it can be administered once daily [7].

The Sanford Guide for Antimicrobial Therapy recommends that the dosing regimens of ceftriaxone be 1–2 g once or twice daily [8]. In fact, a 2 g once daily administration remains a frequently used standard dosing regimen [9,10]. However, there are no studies comparing the effectiveness and safety of either 1 g twice daily or 2 g once daily administration for the treatment of aspiration pneumonia.

In the present study, we retrospectively evaluated the effectiveness and safety of 1 g twice daily and 2 g once daily of ceftriaxone for the treatment of aspiration pneumonia.

## 2. Results

### 2.1. Patients

A total of 61 patients met the criteria during the study period (group 1, *n* = 33; group 2, *n* = 28). Table 1 shows the demographics and clinical characteristics of the two groups. Demographics such as sex, age, and body weight showed no difference between the two groups. The duration of ceftriaxone therapy did not differ between the two groups. The laboratory data regarding inflammatory markers and hepatic functions were comparable between the two groups (group 1 vs. group 2, white blood cells (WBCs), 9.05 × 10^3^/μL vs. 10.26 × 10^3^/μL, *p* = 0.3575; body temperature, 37.8 °C vs. 37.8 °C, *p* = 0.8535; C-reactive protein (CRP), 6.15 mg/dL vs. 7.85 mg/dL, *p* = 0.6883; aspartate aminotransferase (AST), 28 U/L vs. 25 U/L, *p* = 0.3974; alanine transaminase (ALT), 21 U/L vs. 18 U/L, *p* = 0.4268; alkaline phosphatase (ALP), 176 U/L vs. 193 U/L, *p* = 0.4729; and γ-glutamyl transpeptidase (γ-GTP), 31 U/L vs. 39 U/L, *p* = 0.7523). Among the included patients, 86% had mild-to-moderate aspiration pneumonia, with a median oxygen saturation (SpO_2_) of 95.5% and a range of 89–100% before ceftriaxone administration. There were no significant differences in the severity of pneumonia and SpO_2_ between the two groups (A-DROP, *p* = 0.6751; PSI, *p* = 0.8099; SpO_2_, 95.5% vs. 96%, *p* = 0.8649). Additionally, the percentage of patients treated with oxygen supplementation was similar between the two groups (45.5% vs. 42.9%, *p* = 0.8387). The investigation items regarding comorbidity and medication were not significantly different between the two groups (malignancy, 33.3% vs. 39.3%, *p* = 0.6295; hepatic disease, 12.1% vs. 3.6%, *p* = 0.2251; heart disease, 9.1% vs. 21.4%, *p* = 0.1757; cerebrovascular disease, 33.3% vs. 21.4%, *p* = 0.3014; kidney disease, 12.1% vs. 14.3%, *p* = 0.8029; steroid, 9.1% vs. 10.7%, *p* = 0.8320; and immunosuppressant, 0% vs. 7.1%, *p* = 0.1185). The most frequently detected isolate from the study patients was *Streptococcus pneumoniae* (48.5% vs. 46.4%), followed by *Staphylococcus* spp. (18.2% vs. 3.6%), *Klebsiella pneumoniae* (3.0% vs. 7.1%), and *Escherichia coli* (0% vs. 3.6%). The numbers of patients in groups 1 and 2 for whom sputum culture was not performed were 10 (30.3%) and 11 (39.3%), respectively. All isolates were susceptible against ceftriaxone according to the Clinical and Laboratory Standards Institute (CLSI) criteria (minimum inhibitory concentration (MIC): *S. pneumoniae*, 0.12–0.25 mg/L; *Staphylococcus* spp., ≤4 mg/L; *Klebsiella pneumoniae*, ≤1 mg/L; *Escherichia coli*, 0.5 mg/L) [11].

### 2.2. Clinical Effectiveness

Data regarding clinical effectiveness are presented in Table 2. Regarding the clinical responses, 28 of 33 patients in group 1 and all patients in group 2 achieved clinical success at the end of the ceftriaxone therapy (group 1 vs. group 2: clinical cure, 72.7% vs. 75.0%; clinical improvement, 12.1% vs. 25.0%), with significant between-group difference (clinical success, 84.8% vs. 100%, *p* = 0.0316). The percentage of patients with body temperature < 37.0 °C at the end of the therapy was 45.5% and 78.3% in groups 1 and 2, respectively. The percentage of patients with CRP levels < 60% of the baseline at the end of the therapy was 25.0% and 60.7% in groups 1 and 2, respectively. These achievement rates of group 2 were significantly higher than those of group 1 (body temperature, *p* = 0.01; CRP, *p* < 0.01). The survival rates in groups 1 and 2 were 93.9% and 100%, respectively (*p* = 0.1853).

**Figure 1:**
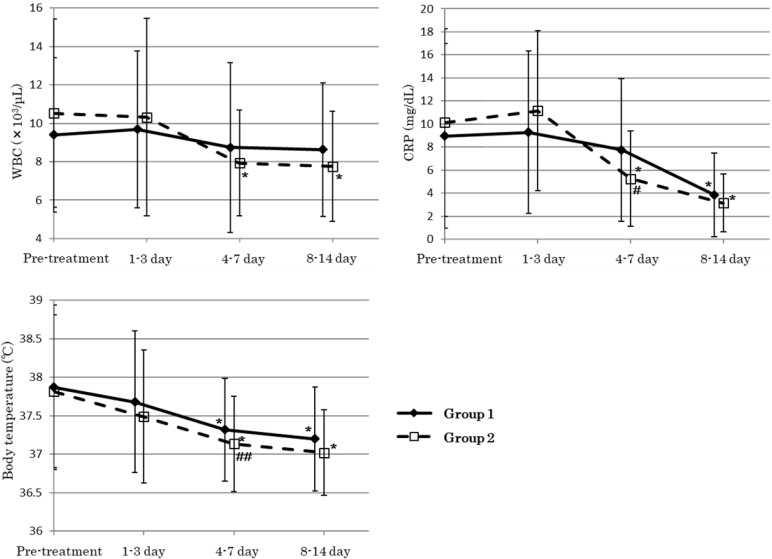
Time-dependent changes in WBC, CRP, and body temperature during and after initiation of ceftriaxone therapy. Data are presented as the mean ± SD. *: *p* < 0.05 (vs. pre-treatment: Dunnett’s test); #: *p* < 0.05 (vs. group 1: unpaired t-test); ##: *p* = 0.0523 (vs. group 1: unpaired *t*-test). Group 1, *n* = 33; group 2, *n* = 28. CRP, c-reactive protein; SD, standard deviation; WBC, white blood cell.

The change in WBC, CRP, and body temperature for 2 weeks after the initiation of ceftriaxone therapy are shown in Figure 1. Group 1 showed a reduction in WBC counts; however, there were no significant differences between the pre-treatment counts and the counts after each timing. The CRP values in group 1 significantly decreased 8–14 days after the initiation of ceftriaxone therapy. Alternatively, group 2 showed significant improvement in WBC and CRP from days 4–7 and 8–14 days after the initiation of ceftriaxone therapy, compared with the pre-treatment values. Both groups showed significant improvements in body temperature on days 4–7 and 8–14 compared with the pre-treatment values. However, CRP value and body temperature on days 4–7 in group 2 were significantly lower than those in group 1 (group 1 vs. group 2: CRP, 7.7 ± 6.2 mg/dL vs. 5.3 ± 4.1 mg/dL, *p* < 0.05; body temperature, 37.3 ± 0.7 °C vs. 37.1 ± 0.6 °C, *p* = 0.0523).

### 2.3. Safety Evaluation

The safety data are shown in Table 3 and Table 4. AST, ALT, ALP, and γ-GTP levels showed no significant differences pre- and post-treatment (Table 3). Several patients had abnormal AST, ALT, and γ-GTP levels after ceftriaxone therapy; one, three, and three patients had abnormalities in AST, ALT, and γ-GTP levels, respectively. The percentage of patients diagnosed with choleliths during ceftriaxone therapy in group 1 was higher than that in group 2 (group 1 vs. group 2, 31.3% vs. 9.1%, *p* = 0.174, Table 4).

## 3. Discussion

The present study compared the effectiveness and safety of ceftriaxone administered 1 g twice daily or 2 g once daily for patients with aspiration pneumonia. The results of this study suggest that 2 g once daily administration may prevent treatment failure, while limiting adverse events, compared with 1 g twice daily administration.

Previous clinical studies involving adult and pediatric patients with severe bacterial infections have highlighted the efficacy of 2 g once daily administration of ceftriaxone [12,13]. In a comparison of ceftriaxone regimens in acute cholangitis, patients receiving 2 g once daily administration recovered to achieve normal WBC values and showed a defervescence below 37 °C early after ceftriaxone administration, as compared with those receiving 1 g twice daily administration [14]. In the present study of patients with aspiration pneumonia, 2 g once daily administration reduced the inflammatory markers early when compared with 1 g twice daily administration. Moreover, the once daily dosing made it possible to treat 20% of the severely ill inpatients as outpatients and allowed substantial cost savings [15]. Therefore, our findings might have contributed to the simplification of complex regimens in hospital settings and the introduction of a 2 g once daily regimen in outpatient departments.

From the viewpoint of pharmacokinetics/pharmacodynamics (PK/PD), beta-lactams increase clinical efficacy by increasing the frequency of administrations [16]. In fact, clinical studies reported the effectiveness of ceftriaxone twice daily [17,18]. Ceftriaxone exerts its bactericidal effect in a time-dependent manner, and the time spent above the MIC (%TAM) is an important PK/PD parameter for estimating clinical efficacy. Some PK/PD studies suggest the use of 1 g twice daily administration; however, this suggestion is only based on the achievement of PK/PD target, not safety [19]. Further studies considering the achievement of PK/PD target and safety is needed in adult patients. Two g once daily administration reportedly produces serum concentrations 10 to 100 times higher than the MICs of clinical isolates over 24 h [15]. Moreover, ceftriaxone is highly protein-bound at low concentrations since its bonding rate is concentration-dependent, while this binding decreases at higher concentrations [20]. Recently, the time-course changes of bacterial killing have been examined using a hollow-fiber infection model with ceftriaxone-susceptible *S. aureus* [21]. The findings revealed that 1 g twice daily administration initially resulted in increased bacterial growth within the first 12 h, but then there was a 1-log reduction in the bacterial burden; however, this effect lessened after 36 h of treatment. Alternatively, a 2 g once daily administration achieved a 1-log 10 reduction in the bacterial burden over 24 h, and this was maintained until 72 h. Another dynamic in vitro study using a time-kill assay with ceftriaxone-susceptible *S. aureus* also showed that the 2 g once daily administration achieved bacterial killing within the first 24 h [22]. These findings may be one of the reasons to corroborate early improvement with 2 g once daily administration. However, the present study was not designed to measure the blood concentration of ceftriaxone and cultivate sputum from patients after completion of ceftriaxone administration.

Estimates of adverse reactions to ceftriaxone range between 1 and 10% [23]. To date, the safety of 2 g once daily and 1 g twice daily administration has been investigated in the US and Switzerland [12,24]. These studies showed that there was no significant difference in number or severity of adverse reactions between 2 g once daily and 1 g twice daily administration. Similarly, our study demonstrates that the rate of adverse reactions was similar between the two regimens. These results suggested no concern for an increase in adverse reactions from a 2 g once daily administration, with no association with the incidence of adverse reactions ang different populations. However, the presence of choleliths has been reported in 21.4% of adults who received ceftriaxone [25]. A literature review of adult patients has reported that the 2 g daily administration of ceftriaxone is associated with the occurrence of choleliths [26]. In the present study, the presence of choleliths was 22.2% in all patients who received 2 g daily of ceftriaxone. Moreover, the presence of cholelith in the patients receiving 1 g twice daily was higher than in those receiving 2 g once daily. Therefore, the 2 g once daily administration clearly benefitted patients in terms of toxicity avoidance. However, further studies are needed to reveal this difference since the mechanism for cholelith formation by ceftriaxone remains unclear.

Previous studies have shown that anaerobes are involved in 9.8% of patients with healthcare-associated pneumonia and 15.7% of patients with community-acquired pneumonia [27,28], while anaerobes are isolated in 29.6% of patients with lung abscess, which is secondary disease of aspiration pneumonia [29]. In this study, all patients were diagnosed as pneumonia without lung abscesses. Therefore, it seems that our study population with aspiration pneumonia without lung abscesses are at low risk due to anaerobe. However, it is necessary to be strongly aware of anaerobic bacteria in patients who are resistant to ceftriaxone treatment or who have an unfavorable clinical course with ceftriaxone treatment.

*S. pneumoniae* and *Haemophilus influenzae* are the two most common aerobic isolates associated with community-acquired aspiration pneumonia [3]. Ceftriaxone has a great in vitro activity against penicillin-resistant pneumococci and *H. influenzae* including ampicillin-resistant strains [30,31]. Furthermore, in this study, these pathogens represented a large percentage of isolates.

Our study has several limitations that are common to previous clinical studies. As a strong limitation, the bacteriological origins of aspiration pneumonia were not identified in this study. Particularly, it was unclear whether anaerobes were a causative pathogen or not, since anaerobic culturing was not performed in this study. Moreover, cultivating tests to ensure the microbiological efficacy were not performed after ceftriaxone treatment, since this is a retrospective study. In the future, further well-designed studies or murine infection models are needed to validate the microbiological efficacy. Next, this was a non-randomized, single-center retrospective study. However, there was no difference in clinical characteristics between the two regimens. Finally, 33 and 28 patients were treated with 1 g twice daily and 2 g once daily of ceftriaxone throughout the entire period. Although the quality of the study is limited by its small sample size, its statistically significant results suggest that these findings hold up with those from larger sample sizes.

## 4. Patients and Methods

### 4.1. Patient Population

We retrospectively reviewed the medical records of patients receiving ceftriaxone for the treatment of aspiration pneumonia at the Mie University Hospital from January 2015 to September 2021. The following patients were excluded from the study: (1) patients who were less than 15 years old, (2) patients receiving ceftriaxone for less than 2 days, (3) and patients with lung abscesses. The study was reviewed and approved by the ethics committee of the Mie University Hospital (No. H2021-229).

### 4.2. Diagnosis and Dosing Regimens

According to previously published international guidelines [32,33], pneumonia was diagnosed by a constellation of clinical features, and a demonstrable infiltrate by chest radiography or other imaging techniques. Aspiration pneumonia was defined as pneumonia among patients with at least one aspiration-related risk factor, including aspiration episodes, dysphagia, disturbed consciousness, neuromuscular diseases, cerebrovascular diseases, tube feeding, or bedridden status [34]. The severity of pneumonia was evaluated using age, dehydration, oxygen saturation, orientation, blood pressure (A-DROP) [35], and pneumonia severity index (PSI) [36] scores. In addition, identification tests for isolated organisms from sputum samples were conducted in the laboratory department of Mie University Hospital. Patients were divided into two groups according to their dosing regimens. Group 1 patients received 1 g ceftriaxone twice daily whereas group 2 patients received 2 g ceftriaxone once daily.

### 4.3. Data Collection

At least 3 days before the start of the ceftriaxone therapy, treatment data, including patient demographics, hospitalization history, and laboratory data, were retrospectively collected through an electronic chart review. Additionally, clinical outcomes and ceftriaxone-related adverse events were recorded for each patient.

### 4.4. Clinical Effectiveness

Clinical responses to ceftriaxone therapy were classified as clinical cure, clinical improvement, and treatment failure [37], each of which was judged by physicians. Clinical cure was defined as the resolution of the baseline clinical signs and symptoms of pneumonia with improvement or lack of progression of radiographic findings. Clinical improvement was defined if a clinical cure was not achieved, but any improvements in the baseline signs and symptoms of pneumonia were observed at the end of the therapy. Treatment failure was defined as the persistence or progression of the baseline signs and symptoms of pneumonia or the administration of another effective antimicrobial due to clinical improvement from the treatment with either of the two antimicrobials of interest. Clinical success included clinical cure and improvement. Data regarding inflammatory markers, including baseline values of body temperature, WBC, and CRP, were collected at least 3 days before initiation and for 2 weeks after initiation of the ceftriaxone therapy for evaluating clinical effectiveness. We also evaluated the percentages of patients who reached a body temperature < 37.0 °C and CRP < 60% of the baseline level. Survival rates were defined as being alive 14 days after initiation of ceftriaxone therapy.

### 4.5. Safety Evaluation

The abnormality of laboratory data was evaluated using Common Terminology Criteria for Adverse Events (CTCAE) version 5.0. Abnormality was defined as follows: AST, >2 times the upper baseline or >30 U/L; ALT, >2 times the upper baseline or >42 U/L; ALP, >2.5 times the upper baseline or >322 U/L; and γ-GTP, 2 times the upper baseline or >50 U/L (male) or >32 U/L (female). In addition, the presence of choleliths was evaluated using the chart records of abdominal computed tomographic scans during the ceftriaxone therapy.

### 4.6. Statistical Analysis

The data regarding the clinical characteristics of patients were expressed as the median values (minimum-maximum). Statistical significance was evaluated using the Chi-square test for categorical data and unpaired *t*-test for continuous data. In addition, multiple comparisons of inflammatory markers were performed using Dunnett’s test. Statistical analysis was performed using JMP, version 10.0 (SAS, Tokyo, Japan). A *p*-value < 0.05 was considered statistically significant.

## 5. Conclusions

In conclusion, the present study demonstrated that a 2 g once daily administration of ceftriaxone improved clinical responses and reduced inflammatory markers early in patients with mild-to-moderate aspiration pneumonia, while limiting adverse events. Hence, the 2 g once daily administration appears to be a simple regimen that is adequate for the treatment of inpatients with mild-to-moderate aspiration pneumonia which might not be heavily involved by anaerobes.

## Figures and Tables

**Table 1:** Clinical Characteristics of Registered Patients.

		Group 1	Group 2	*p*-Value
Sex (male/female)		21/12	21/7	0.3396
Age (years)		73 (17–90)	72 (41–86)	0.3020
Body weight (kg)		55.8 (36.8–103.6)	52.9 (29.0–81.1)	0.3389
Duration of therapy (day)		6 (3–17)	6 (3–15)	0.6093
WBC (×10^3^/μL)		9.05 (3.52–18.45)	10.26 (1.92–22.31)	0.3575
Albumin (g/dL)		3.3 (2.1–4.3)	3.9 (1.9–5.5)	0.6958
Serum creatinine (mg/dL)		0.82 (0.25–3.71)	1.00 (0.38–12.05)	0.1018
eGFR (mL/min/1.73 m^2^)		66.3 (15.1–289.3)	55.6 (3.9–135.1)	0.6646
Blood urea nitrogen (mg/dL)		18.6 (2.9–66.2)	17.6 (7.0–87.3)	0.5334
AST (U/L)		28 (16–795)	25 (12–118)	0.3974
ALT (U/L)		21 (8–519)	18 (4–74)	0.4268
ALP (U/L)		176 (53–508)	193 (61–908)	0.4729
γ-GTP (U/L)		31 (10–338)	39 (14–200)	0.7523
Total bilirubin (mg/dL)		0.7 (0.2–3.8)	0.7 (0.3–1.5)	0.3250
A-DROP (%, *n*)				0.6751
	0–1	48.5, 16/33	57.1, 16/28	
	2	39.4, 13/33	28.6, 8/28	
	3	12.1, 4/33	14.3, 4/28	
	4–5	0, 0	0, 0	
PSI (%, *n*)				0.8099
	I–II	24.2, 8/33	14.3, 4/28	
	III	12.1, 4/33	14.3, 4/28	
	IV	45.5, 15/33	50.0, 14/28	
	V	18.2, 6/33	21.4, 6/28	
SpO_2_ (%)		95.5 (89–100)	96 (91–99)	0.8649
Oxygen supplemantation (%, *n*)		45.5, 15/33	42.9, 12/28	0.8387
Body temperature (°C)		37.8 (36.4–40.6)	37.8 (36.7–39.8)	0.8535
CRP (mg/dL)		6.15 (0.04–26.45)	7.85 (0.20–24.70)	0.6883
Comorbidity (%, *n*)				
	Malignancy	33.3, 11/33	39.3, 11/28	0.6295
	Hepatic disease	12.1, 4/33	3.6, 1/28	0.2251
	Heart disease	9.1, 3/33	21.4, 6/28	0.1757
	Cerebrovascular disease	33.3, 11/33	21.4, 6/28	0.3014
	Kidney disease	12.1, 4/33	14.3, 4/28	0.8029
Medication (%, *n*)				
	Steroid	9.1, 3/33	10.7, 3/28	0.8320
	Immunosuppressant	0, 0/33	7.1, 2/28	0.1185

The Chi-square test was used for categorical data and the unpaired *t*-test for continuous data. All data, except sex, are shown as the median (minimum-maximum). Group 1, patients receiving ceftriaxone 1 g twice daily (*n* = 33); group 2, patients receiving ceftriaxone 2 g once daily (*n* = 28). ALT, alanine aminotransferase; ALP, alkaline phosphatase; AST, alanine aminotransferase; CRP, c-reactive protein; eGFR, estimated glomerular filtration rate; γ-GTP, γ-glutamyl transpeptidase; PSI, pneumonia severity index; SpO_2_, oxygen saturation; WBC, white blood cell.

**Table 2:** Clinical effectiveness of registered patients.

			Group 1	Group 2	*p*-Value
At the end of the ceftriaxone therapy (%)					0.0316
	Clinical success		84.8 (28/33)	100 (28/28)	
		Cure	72.7 (24/33)	75.0 (21/28)	
		Improvement	12.1 (4/33)	25.0 (7/28)	
	Clinical failure		15.2 (5/33)	0 (0)	
BT < 37.0 °C (%)			45.5 (15/33)	78.3 (18/23)	0.0141
CRP < 60% (%)			25.0 (8/32)	60.7 (17/28)	0.0051
Survival rate (%)			93.9 (31/33)	100 (28/28)	0.1853

The Chi-square test was used for categorical data. Group 1, patients receiving ceftriaxone 1 g twice daily (*n* = 33); group 2, patients receiving ceftriaxone 2 g once daily (*n* = 28). BT, body temperature; CRP, C-reactive protein.

**Table 3:** Safety data at pre-treatment and post-treatment.

		Pre-Treatment	Post-Treatment	*p*-Value
Group 1	AST (U/L)	55.3 ± 135.6	36.7 ± 22.0	0.3831
	ALT (U/L)	37.8 ± 88.5	54.2 ± 116.5	0.7975
	ALP (U/L)	211.6 ± 120.2	235.2 ± 181.8	0.2302
	γ-GTP (U/L)	51.7 ± 70.6	99.3 ± 127.0	0.1864
Group 2	AST (U/L)	33.1 ± 24.1	31.8 ± 19.9	0.6938
	ALT (U/L)	24.1 ± 17.8	27.6 ± 21.1	0.3396
	ALP (U/L)	240.9 ± 181.3	230.3 ± 165.8	0.5011
	γ-GTP (U/L)	57.7 ± 51.6	61.3 ± 57.5	0.3829

An unpaired *t*-test was used for continuous data. Group 1, patients receiving ceftriaxone 1 g twice daily (*n* = 33); group 2, patients receiving ceftriaxone 2 g once daily (*n* = 28). ALT, alanine aminotransferase; ALP, alkaline phosphatase; AST, alanine aminotransferase; γ-GTP, γ-glutamyl transpeptidase.

**Table 4:** Safety data of the registered patients.

	Group 1	Group 2	*p*-Value
Increased AST level (%)	3.0 (1/33)	0 (0/28)	0.3530
Increased ALT level (%)	6.1 (2/33)	3.6 (1/28)	0.6542
Increased ALP level (%)	0 (0/26)	0 (0/25)	-
Increased γ-GTP level (%)	8.3 (2/24)	5.0 (1/20)	0.6623
Presence of choleliths (%)	31.3 (5/16)	9.1 (1/11)	0.1740

The Chi-square test was used for categorical data. Group 1, patients receiving ceftriaxone 1 g twice daily (*n* = 33); group 2, patients receiving ceftriaxone 2 g once daily (*n* = 28). ALT, alanine aminotransferase; ALP, alkaline phosphatase; AST, alanine aminotransferase; γ-GTP, γ-glutamyl transpeptidase.

## Data Availability

All data are applicable in the paper.
